# Quantification and classification of lumbar disc herniation on axial magnetic resonance images using deep learning models

**DOI:** 10.1007/s11547-025-01996-y

**Published:** 2025-03-24

**Authors:** Elzat Elham-Yilizati Yilihamu, Jun Shang, Zhi-Hai Su, Jin-Tao Yang, Kun Zhao, Hai Zhong, Shi-Qing Feng

**Affiliations:** 1 Orthopedic Research Center of Shandong University & Advanced Medical Research Institute, Shandong University, Jinan, 250000 China; 2 Qilu Hospital of Shandong University, Shandong University, Jinan, 250000 China; 3 Renci Hospital of Xuzhou Medical University, Xuzhou, 221000 China; 4 Fifth Affiliated Hospital of Sun Yat-Sen University, Spinal Surgery, Zhuhai, 519000 Guangdong China; 5 Medical Research Department of Jiangsu Shiyu Intelligent Medical Technology Co., Nanjing, 210000 China; 6 The Second Hospital of Shandong University, Cheeloo College of Medicine, Shandong University, Jinan, 250000 China; 7 Tianjin Medical University General Hospital, Tianjin, 300041 China

**Keywords:** Lumbar disc herniation, Deep learning, MRI, Low back pain

## Abstract

**Purpose:**

Application of a deep learning model visualization plugin for rapid and accurate automatic quantification and classification of lumbar disc herniation (LDH) types on axial T2-weighted MRIs.

**Methods:**

Retrospective analysis of 2500 patients, with the training set comprising data from 2120 patients (25,554 images), an internal test set covering data from 80 patients (784 images), and an external test set including data from 300 patients (3285 images). To enhance implementation, this study categorized normal and bulging discs as a grade without significant abnormalities, defining the region and severity grades of LDH based on the relationship between the disc and the spinal canal. The automated detection training and validation process employed the YOLOv8 object detection model for target area localization, the YOLOv8-seg segmentation model for disc recognition, and the YOLOv8-pose keypoint detection model for positioning. Finally, the stability of the detection results was verified using metrics such as Intersection over Union (IoU), mean error (ME), precision (P), F1 score (F1), Kappa coefficient (kappa), and 95% confidence interval (95%CI).

**Results:**

The segmentation model achieved an mAP50:95 of 98.12% and an IoU of 98.36% in the training set, while the keypoint detection model achieved an mAP50:95 of 93.58% with a mean error (ME) of 0.208 mm. For the internal and external test sets, the segmentation model’s IoU was 97.58 and 97.49%, respectively, while the keypoint model’s ME was 0.219 mm and 0.221 mm, respectively. In the quantification validation of the extent of LDH, P, F1, and kappa were measured. For LDH classification (18 categories), the internal and external test sets showed *P* = 81.21% and 74.50%, F1 = 81.26% and 74.42%, and kappa = 0.75 (95%CI 0.68, 0.82, *p* = 0.00) and 0.69 (95%CI 0.65, 0.73, *p* = 0.00), respectively. For the severity grades of LDH (four categories), the internal and external test sets showed *P* = 92.51% and 90.07%, F1 = 92.36% and 89.66%, and kappa = 0.88 (95%CI 0.80, 0.96, *p* = 0.00) and 0.85 (95%CI 0.81, 0.89, *p* = 0.00), respectively. For the regions of LDH (eight categories), the internal and external test sets showed *P* = 83.34% and 77.87%, F1 = 83.85% and 78.21%, and kappa = 0.77 (95%CI 0.70, 0.85, *p* = 0.00) and 0.71 (95%CI 0.67, 0.75, *p* = 0.00), respectively.

**Conclusion:**

The automated aided diagnostic model achieved high performance in detecting and classifying LDH and demonstrated substantial consistency with expert classification.

**Electronic supplementary material:**

The online version of this article (10.1007/s11547-025-01996-y) contains supplementary material, which is available to authorized users.

## Introduction

In 2017, approximately 577 million people worldwide were reported to have low back pain, leading to high rates of absenteeism and disability, with lumbar disc herniation (LDH) being considered the most common cause [1, 2]. When diagnosing such conditions, MRI has become the preferred modality for imaging the lumbar spine due to its excellent performance in soft tissue imaging [3].

Although the LDH classification method recommended in the “Lumbar disc nomenclature: version 2.0” jointly released by the American Society of Spine Radiology (ASSR), American Society of Neuroradiology (ASNR), and North American Spine Society (NASS) has a certain grade of validity [4], the actual operation often involves a large amount of mechanical and repetitive work, which is both cumbersome and time-consuming. More importantly, the diagnostic results lack standardized and quantitative evaluation criteria [5].

To more effectively guide the diagnosis of lumbar spine diseases, researchers have recently begun to apply deep learning (DL) techniques to the diagnosis of LDH. Hallinan et al. [6] and Lim et al. [7] reported methods for the automatic classification and diagnosis of lumbar spinal stenosis. Zhang et al. [8] implemented automatic determination of grades within the MSU classification system [9] using a classification model. Although these studies have achieved some success in automating diagnosis, they still fail to meet the needs for quantitative analysis of the severity of the lesion area, whether using binary or four-class categorizations [10, 11]. Additionally, doctors still need to spend a significant amount of time verifying the accuracy of the results [12].

To more accurately quantify the degree of lumbar intervertebral disc degeneration, Xuan et al. [13] employed object detection and transfer learning models, successfully quantifying LDH and spondylolisthesis in the sagittal plane with an accuracy of 90.08%. Simultaneously, Li et al. [14] constructed a vertebral body object detection model based on sagittal lumbar MRI to verify the relationship between vertebral bodies and intervertebral discs. Laiwalla et al. [15] established an evaluation model for spinal canal stenosis in the axial MRI model. Previous studies have fully demonstrated the excellent application potential of artificial intelligence techniques in the detection of LDH [16–18]. Despite significant progress in this field, unfortunately, to date, there has not been a complete system that can truly assist the clinical diagnostic process.

In this study, we utilized DL models to accurately locate and quantify the anatomical relationship parameters between intervertebral discs and vertebral bodies. Furthermore, we integrated a DL model into an image reading system and developed a computer-aided diagnosis tool that can visualize and automatically interpret the anatomical features of axial MRIs of intervertebral discs. To verify the practicality and reliability of this system, we conducted in-depth testing on multi-center datasets. The test results showed that the DL model exhibited excellent performance and strong generalization ability on the external test set, fully demonstrating the enormous potential of this model in the field of aided diagnosis.

## Materials and methods

### Patient population

This retrospective study was conducted in centers across four different provinces, involving 12 different MRI devices, including Tianjin Medical University General Hospital (training, validation and internal test set), the Second Hospital of Shandong University, Xuzhou Renci Hospital, and the Fifth Affiliated Hospital of Sun Yat-sen University (external test set). The inclusion criteria were as follows: (1) age 18 years and above; (2) patients who had undergone lumbar spinal MRI. The exclusion criteria were: (1) patients who had undergone lumbar surgical treatment; (2) patient data unrelated to orthopedic diseases; (3) MRI imaging quality not meeting the orthopedists’ image reading standards. Data were randomly extracted from January 2015 to December 2022. This study was retrospective; patient information was de-identified, and the risk was minimal; therefore, all four centers waived the consent requirement.

### Data distribution in training, validation, internal test set, and external test sets

The training set included 25,554 axial lumbar MRIs from 2120 patients. Among these images, 12,161 MRIs were specifically used for intervertebral disc segmentation training, while 22,297 were used for keypoint detection training of the articular process and spinous process regions. During training, these image data were randomly divided into a training set and a validation set at a ratio of 9:1. The internal test set consisted of 784 axial lumbar MRIs from 80 patients, while the external test set covered 3285 MRIs from 300 patients.

### Image processing

**Fig. 1:**
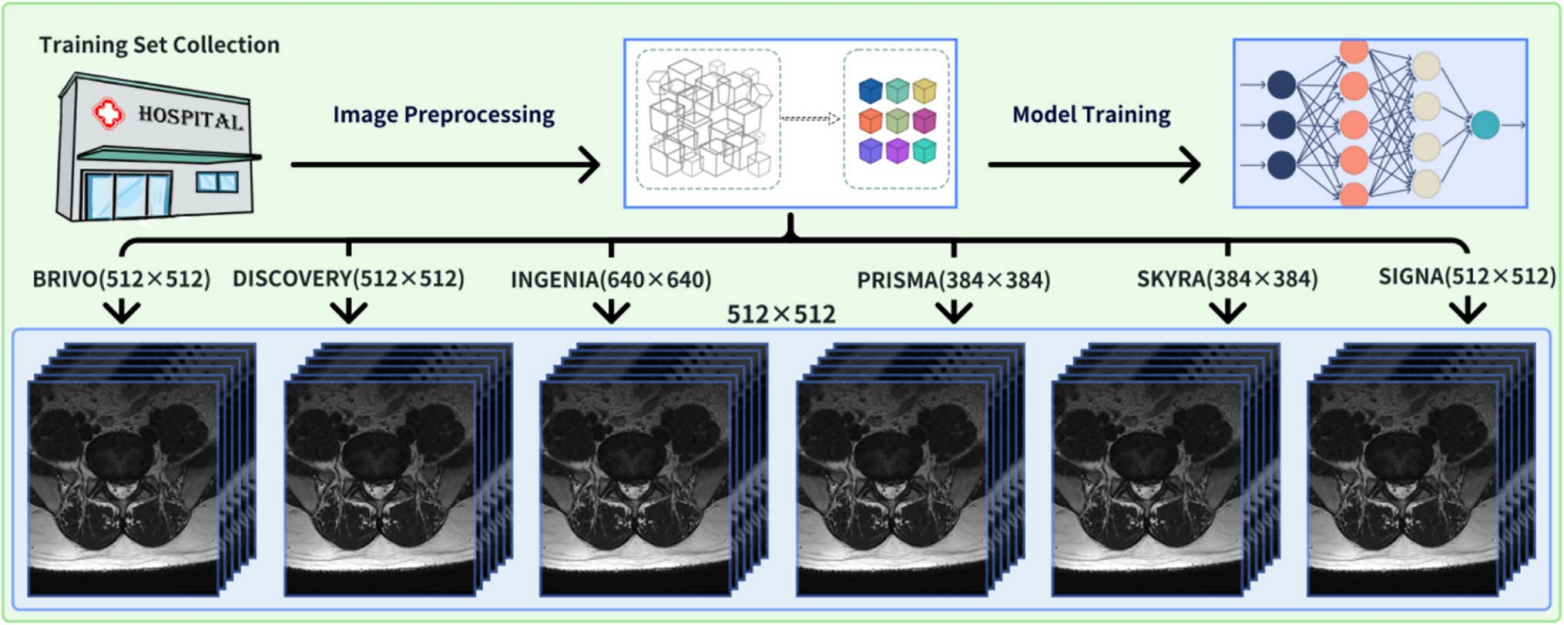
Training set allocation and processing flowchart

To minimize the impact of image differences between different devices on data boundary consistency, this study employed various strategies to process images generated by different MRI devices. Image sizes were standardized and image enhancement techniques were applied to ensure stable training results were achieved. Specifically, all images of different sizes in the training set were uniformly adjusted to 512 × 512 pixels, and parameters such as grayscale values and contrast were adjusted to enhance the images, thereby improving the model’s ability to recognize images from different MRI devices. Before prediction, all images in the validation set were uniformly adjusted to 512 × 512 pixels (Fig. 1).

### Data annotation

To ensure the accuracy and consistency of data annotation in axial lumbar MRIs, the uniformly sized MRIs were annotated by K.Z, an orthopedist with 8 years of clinical experience, and Z.S, an orthopedist with 4 years of clinical experience, using LabelMe (v3/CSAIL, MIT/USA) software on the training set. When opinions differed, H.Z, a radiologist with 20 years of clinical experience, provided authoritative assessments to ensure the accuracy of the annotated data.

We accurately annotated the segmentation of the lumbar intervertebral disc regions. Through keypoint annotation, the spinal canal was divided into the central canal zone (CCZ), left and right extraforaminal zone (EFZ), left and right subarticular zone (SAZ), and left and right foraminal zone (FZ). To clearly define the ligamentum flavum area at the posterior of the spinal canal, the most anterior vertex of the bilateral articular processes was annotated. Additionally, we annotated the position anterior to the spinous process within the spinal canal, allowing for accurate measurement of the anteroposterior sagittal diameter of the posterior spinal canal ligamentum flavum area. To ensure that the partitions remain perpendicular to the patient’s axial plane, the system automatically moves the partition points to the vertical intersection of the articular process anterior edge line before training and after prediction, ensuring the accuracy and reliability of the partitioning.

### Lumbar disc herniation detection model

**Fig. 2:**
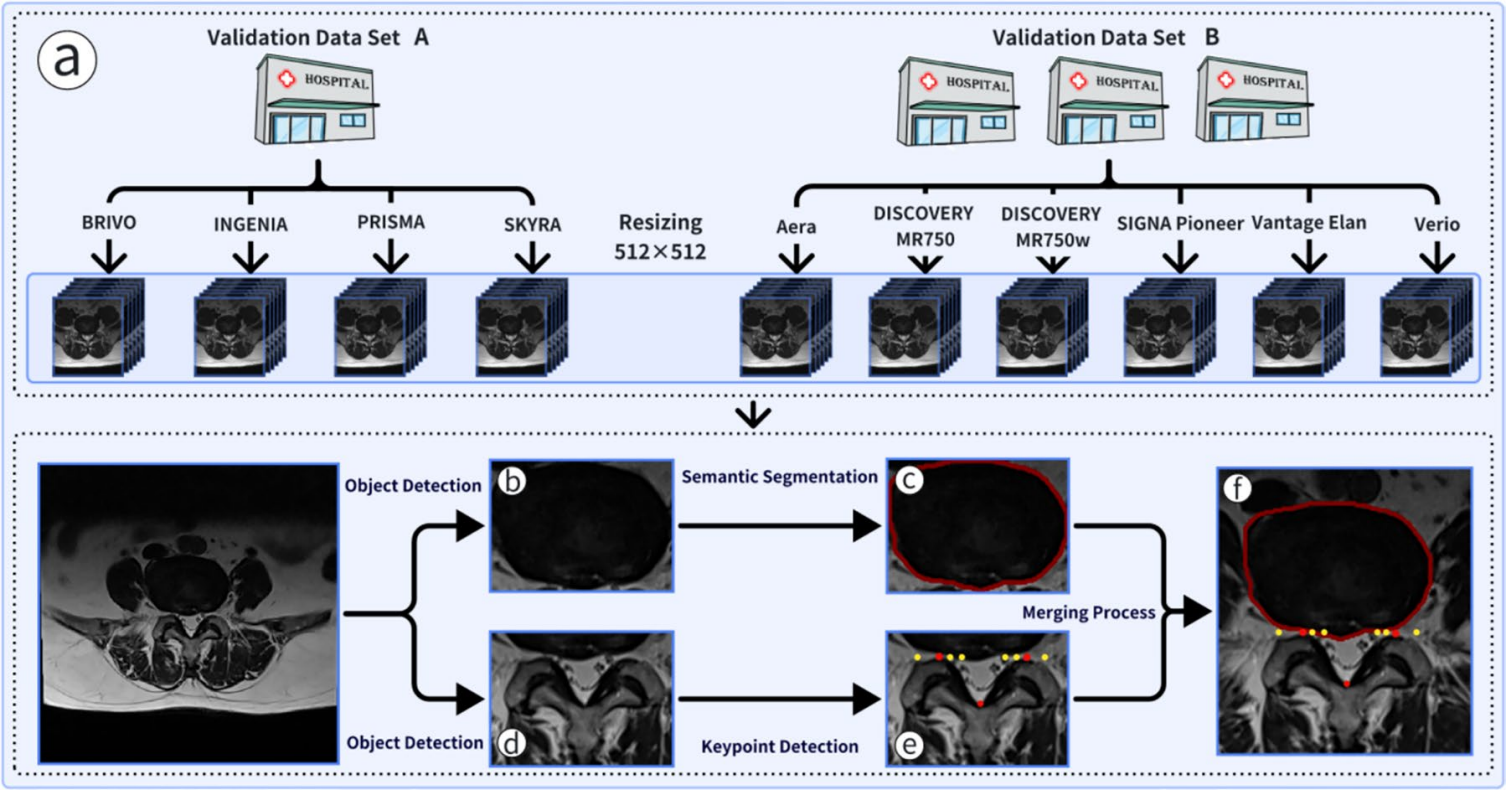
Process of disc herniation localization model. **a** Preparation of test set data, **b** Object detection to locate the intervertebral disc region, **c** Segmentation of the intervertebral disc region, **d** Object detection to locate the articular process and spinous process regions, **e** Keypoint detection to predict the boundaries of the regions, **f** Merging of the segmentation results with the keypoint detection results, awaiting computation

After standardizing the sizes of the internal and external test set data, we utilized multiple DL models to construct a dual-branch multi-stage workflow (Fig. 2a, https://github.com/ElzatElham/LDH-DL-MODEL). Branch 1 primarily involved object detection and semantic segmentation. In the first stage, an object detection model based on YOLOv8 [19] accurately located the intervertebral disc region (Fig. 2b). In the second stage, based on the localization results of the intervertebral disc detection model, a semantic segmentation model using YOLOv8-seg classified each pixel within the intervertebral disc region (Fig. 2c). Branch 2 focused on object detection and keypoint detection. In the first stage, a YOLOv8 object detection model precisely located the articular process and spinous process regions (Fig. 2d). In the second stage, based on the localization results of the spinal canal detection model, a YOLOv8-pose keypoint detection model accurately positioned the edges of the seven zones of the spinal canal (Fig. 2e). To ensure the parallelism of the zones with the patient’s axial position, the system automatically moved the zone edge points to the perpendicular intersection of the anterior edge line of the articular process (Fig. 2f). The entire workflow was compact and efficient, ensuring processing speed while maintaining the accuracy and reliability of the results.

### Localization of the degree and position of intervertebral disc herniation

**Fig. 3:**
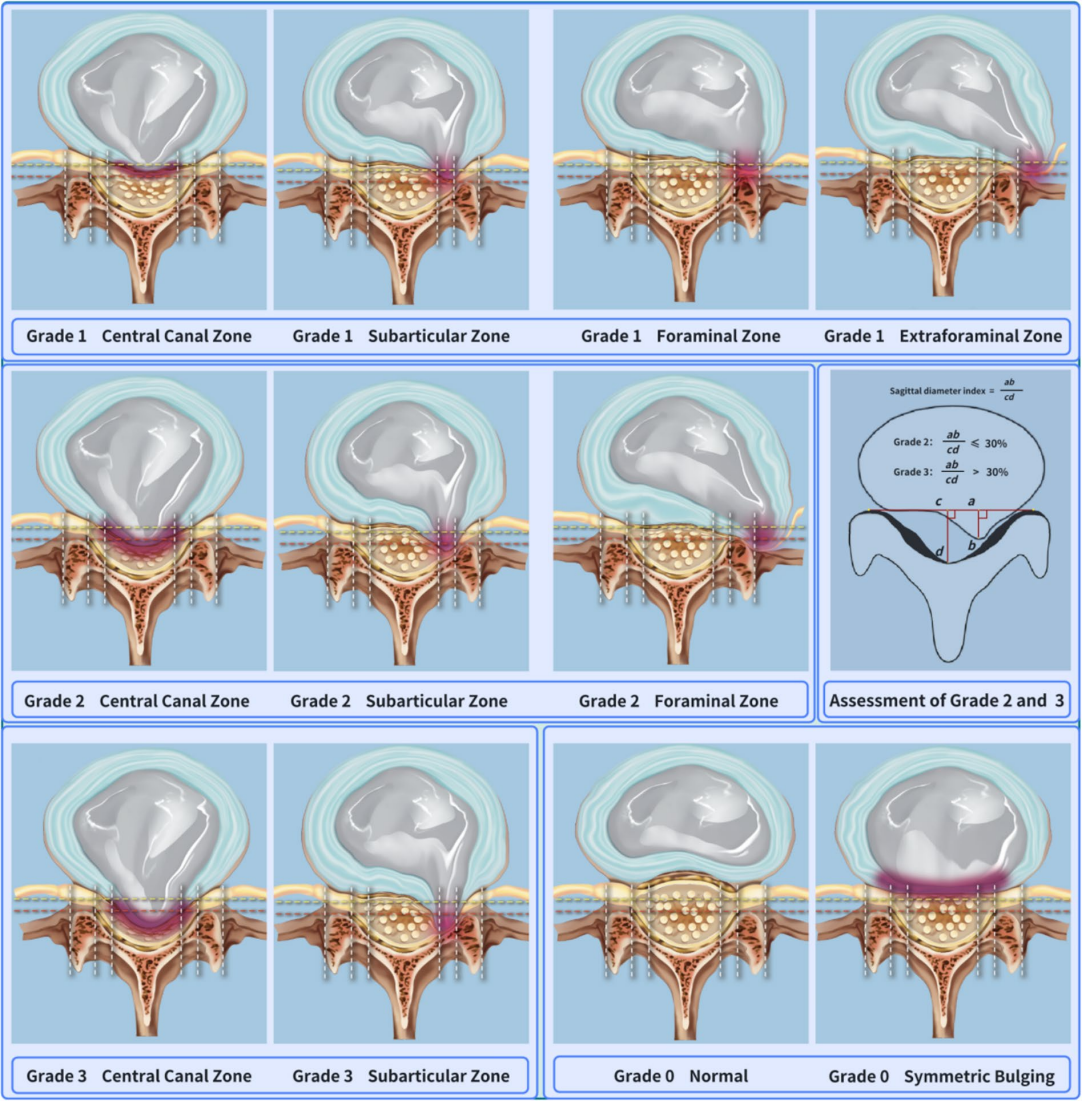
Identification of the anatomical region and grading of herniated intervertebral discs

After the prediction was completed, in order to accurately describe the degree of LDH, we used the results of segmentation and keypoint detection to classify the distribution of the spinal canal. CCZ, SAZ, FZ, and EFZ were used to precisely define the specific location of the LDH, which was graded based on the degree of LDH into the spinal canal (Fig. 3). If the intervertebral disc was in a normal state or the distance to the line connecting the bilateral articular processes was less than 3 mm, it was classified as grade 0; if the herniation was within 3 mm of the line connecting the bilateral articular processes, it was classified as grade 1; if the LDH extended beyond the line connecting the bilateral articular processes (Ligamentum Flavum zone of the central spinal canal) [20], but with an anteroposterior sagittal diameter index ≤ 0.3, it was classified as grade 2; and if the anteroposterior sagittal diameter index was > 0.3, it was classified as grade 3.

### Evaluation of model detection results

**Fig. 4:**
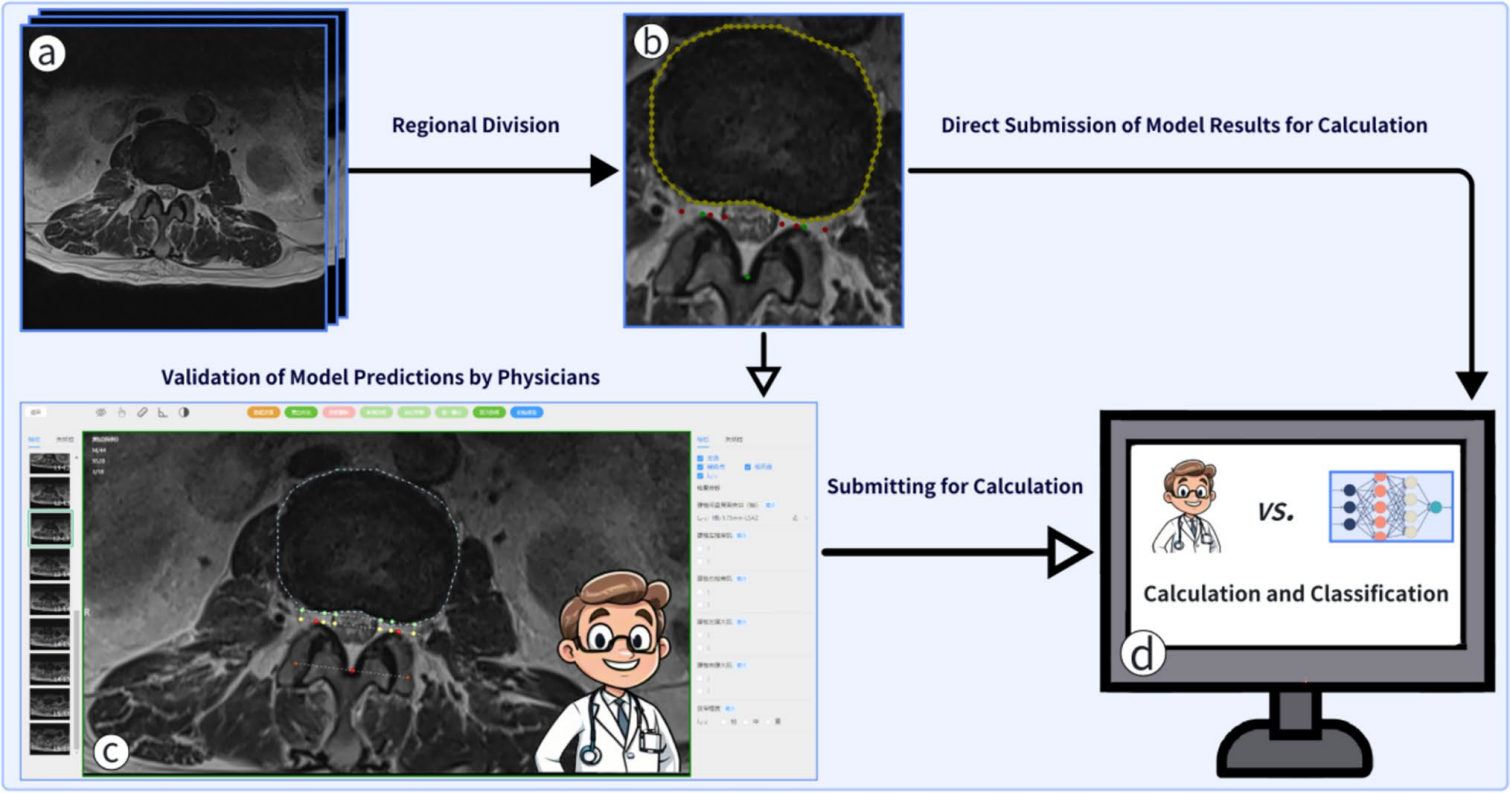
Model results validation and comparative analysis process. **a** Images enter the model. **b** DL model predicts the segmentation and keypoint regions. The solid triangle arrows represent the direct calculation of the model’s original results, while the hollow triangle arrows represent the results being imported into the GPTSP. **c** The orthopedist verifies the data under the GPTSP. **d** The model’s predicted results are compared with the orthopedist’s modified results

After the model made predictions on the test dataset, the results were uniformly imported into the database of the spinal imaging intelligent aided reading system (V1.0SY-GPTSP, SDSY/China). Using the visualization function of GPTSP (as shown in the supplementary material GPTSP.mp4), orthopedic experts E.E with 10 years of clinical experience and J.S with 15 years of clinical experience conducted a comprehensive review of the model’s detection results (Fig. 4). For controversial points in the review, we further consulted the orthopedic expert S.F with 30 years of clinical experience to obtain the final confirmation. Finally, by comparing the data changes before and after the orthopedists’ modifications, we were able to objectively evaluate the reliability of the model.

### Statistical analysis

Continuous variables are presented as mean $\pm$ standard deviation (SD). The consistency between the model’s annotation and manual annotation was evaluated using the Kappa coefficient (kappa). The consistency level of the kappa was defined as follows: when kappa = 0, the consistency was entirely due to chance; when kappa = 1, the two classification results were completely consistent; when kappa ≤ 0.4, the consistency was poor; when 0.4 < kappa ≤ 0.6, the consistency was moderate; when 0.6 < kappa ≤ 0.8, the consistency was high; and when kappa > 0.8, the consistency was excellent. The significance level was *p* < 0.01. Statistical analysis was performed with Python (3.7.16/Python Software Foundation/USA).

## Results

### Patient data set characteristics

The 80 cases in the internal test set comprised 51 females (mean age, 54.86 years ± 14.80 [SD] [range, 28–79 years]) and 29 males (mean age 55.07 years ± 17.74 [SD] [range, 22–99 years]). The external test set comprised 300 cases, with 163 females (mean age 58.94 years ± 14.18 [SD] [range, 25–91 years]) and 137 males (mean age 55.90 years ± 13.69 [SD] [range, 21–89 years]) (Table 1).

**Table 1:** Test dataset and MR Device Information

Dataset	Image number	Age (mean ± standard)	Sex (F/M)	Manufacturer	Model name
Internal test set	100	43.55 ± 12.81	13/7	GE MEDICAL SYSTEMS	BRIVO
	100	59.35 ± 14.47	13/7	SIEMENS	PRISMA
	100	59.55 ± 12.88	16/4	SIEMENS	Skyra
	100	57.30 ± 17.75	9/11	Philips	INGENIA
External test set	250	57.78 ± 10.78	25/25	GE MEDICAL SYSTEMS	DISCOVERY MR750
	250	58.84 ± 14.05	26/24	GE MEDICAL SYSTEMS	DISCOVERY MR750w
	250	61.56 ± 12.43	17/33	GE MEDICAL SYSTEMS	SIGNA Pioneer
	250	63.16 ± 13.49	21/29	SIEMENS	Aera
	250	52.72 ± 14.75	26/24	SIEMENS	Verio
	250	51.26 ± 14.75	22/28	TOSHIBA	Vantage Elan

### Evaluation of Segmentation Model and Keypoint Detection Model

The best-performing models are shown (Table 2). For branch 1 and branch 2, the object detection models achieved mAP50:95 of 96.15% and 97.81%, respectively, the segmentation model achieved mAP50:95 of 98.12% and IoU of 98.36%, and the keypoint detection model achieved mAP50:95 of 93.58% with a mean error (ME) of 0.208 mm (the actual offset distance between points). The results demonstrate that the dual-branch multi-stage workflow exhibits very stable performance in both segmentation and keypoint detection.

**Table 2:** The performance of the DL models in the dual-branch multi-stage workflow

Model name	Model type	Precision (%)	Recall (%)	F1 score (%)	mAP50 (%)	mAP50:95 (%)
Branch 1	Object detection	99.71	99.82	99.76	99.47	96.15
	Semantic segmentation	99.72	99.83	99.76	99.47	98.12
Branch 2	Object detection	99.20	99.91	99.55	99.35	97.81
	Keypoint detection	99.99	99.99	99.99	99.50	93.58

### Model evaluation on the internal test set

In the internal test set, we utilized 80 cases, totaling 400 MRI images. The model achieved an Intersection over Union (IoU) of 97.58% for the localization task, with a mean error of 0.219 mm for keypoint predictions. The classification evaluation for LDH revealed the following results: for the 18 categories (combining severity grades and regions), the accuracy was 82.25%, with precision (*P*) at 81.21% and F1 score at 81.26%, yielding a kappa value of 0.75 (high, 95% CI 0.68, 0.82, *p* = 0.00). For the four categories (severity grades only), the accuracy was 92.50%, with *P* at 92.51% and F1 at 92.36%, resulting in a kappa of 0.88 (excellent, 95% CI 0.80, 0.96, *p* = 0.00). For the eight categories (regions only), the accuracy was 85.00%, with P at 83.34% and F1 at 83.85%, and a kappa of 0.77 (high, 95% CI 0.70, 0.85, *p* = 0.00). These findings demonstrate that the model exhibits a high level of consistency in the localization and quantification of LDH severity and regions, highlighting its significant diagnostic value (see Table 3).

**Table 3:** Classification performance of the DL model and orthopedist in the internal test dataset

Classification		Grade 0		Grade 1		Grade 2		Grade 3	
	Orthopedist	198	(49.50)	–		–		–	
	Model	185	(46.25)	–		–		–	
LEFZ	Orthopedist	–		0	(0.00)	1	(0.25)	0	(0.00)
	Model	–		1	(0.25)	3	(0.75)	0	(0.00)
LFZ	Orthopedist	–		10	(2.50)	–		–	
	Model	–		15	(3.75)	–		-	
LSAZ	Orthopedist	–		17	(4.25)	3	(0.75)	0	(0.00)
	Model	–		23	(5.75)	6	(1.50)	0	(0.00)
CCZ	Orthopedist	–		64	(16.00)	61	(15.25)	6	(1.50)
	Model	–		54	(13.50)	64	(16.00)	8	(2.00)
RSAZ	Orthopedist	–		16	(4.00)	2	(0.50)	0	(0.00)
	Model	–		15	(3.75)	2	(0.50)	0	(0.00)
RFZ	Orthopedist	–		14	(3.50)	–	–		
	Model	–		15	(3.75)	–	–		
REFZ	Orthopedist	–		1	(0.25)	7	(1.75)	0	(0.00)
	Model	–		1	(0.25)	8	(2.00)	0	(0.00)

– indicates that no such classification exists

### Model evaluation on the external test set

In the external test set, we selected 300 cases, totaling 1500 MRI images. The results indicated that the model achieved an Intersection over Union (IoU) of 97.49% for the localization task, with a mean error of 0.221 mm for keypoint predictions. The classification evaluation for LDH revealed the following results: for the 18 categories (combining severity grades and regions), the accuracy was 75.13%, with precision (*P*) at 74.50% and F1 score at 74.42%, yielding a kappa value of 0.69 (indicating high agreement, 95% CI 0.65, 0.73, *p* = 0.00). For the four categories (severity grades only), the accuracy was 90.00%, with *P* at 90.07% and F1 at 89.66%, resulting in a kappa of 0.85 (indicating excellent agreement, 95% CI 0.81, 0.89, *p* = 0.00). For the eight categories (regions only), the accuracy was 78.93%, with *P* at 77.87% and F1 at 78.21%, and a kappa of 0.71 (indicating high agreement, 95% CI 0.67, 0.75, *p* = 0.00). These findings demonstrate that the model maintains high precision in localizing the severity and regions of LDH across multi-center data, validating the diagnostic system’s generalizability and clinical applicability (Table 4).

**Table 4:** Classification performance of the DL model and orthopedist in the external test dataset

Classification		Grade 0		Grade 1		Grade 2		Grade 3	
	Orthopedist	555	(37.00)	–		–		–	
	Model	493	(32.87)	–		–		–	
LEFZ	Orthopedist	–		5	(0.33)	12	(0.80)	0	(0.00)
	Model	–		4	(0.27)	13	(0.87)	0	(0.00)
LFZ	Orthopedist	–		114	(7.60)	–		–	
	Model	–		137	(9.13)	–		–	
LSAZ	Orthopedist	–		82	(5.47)	14	(0.93)	0	(0.00)
	Model	–		95	(6.33)	15	(1.00)	0	(0.00)
CCZ	Orthopedist	–		240	(16.00)	290	(19.33)	23	(1.53)
	Model	–		202	(13.47)	319	(21.27)	36	(2.40)
RSAZ	Orthopedist	–		45	(3.00)	22	(1.47)	0	(0.00)
	Model	–		56	(3.73)	26	(1.73)	1	(0.07)
RFZ	Orthopedist	–		80	(5.33)	–		–	
	Model	–		81	(5.40)	–		–	
REFZ	Orthopedist	–		4	(0.27)	13	(0.87)	1	(0.07)
	Model	–		5	(0.33)	16	(1.07)	1	(0.07)

– indicates that no such classification exists

## Discussion

MRI examination plays an important role in the diagnosis of LDH [21]. However, traditional methods of lumbar MRI analysis involve complex data acquisition processes and heavy workloads, making it difficult to obtain comprehensive and quantitative diagnostic results [22]. To address these issues, we developed an automated aided diagnostic model. The model can efficiently and accurately identify the regions and degrees of LDH in axial MRIs. This DL model can be integrated with image reading system software, allowing doctors to obtain objective and precise diagnostic evidence through visualization and modification of the results.

Previously, researchers have made considerable efforts to standardize the reporting of the location and size of LDH. As early as 1987, Wiltse et al. [23] subdivided the spinal canal into CCZ, SAZ, FZ, and EFZ based on anatomical features. Subsequently, Mysliwiec et al. [9] proposed the MSU classification system, which determines the specific location and size of LDH by measuring the relative position of the herniation to the intra-facet line. However, it is worth noting that the MSU classification system does not differ significantly in essence from the traditional anatomical region division. In practical application, it requires converting the originally-clear anatomical region descriptions into codes such as A, B, C, and 0–1–2–3, which increases the complexity of communication, even without distinguishing left and right, resulting in up to 11 possible combinations. To date, there is no conclusive research confirming the actual clinical guidance value of the MSU classification system. In contrast, this study further validates that the specific location of LDH can be effectively recorded by the boundaries of anatomical landmarks, and the degree of herniation into the spinal canal can be quantified.

With the widespread application of artificial intelligence technology, the automated diagnosis of LDH has achieved preliminary results. The DL models developed by Alomari et al. [24] and Tsai et al. [22] mainly focused on binary and quaternary classification studies, determining the presence or absence of LDH. Zhang et al. [8] adopted the MSU classification system and achieved an accuracy of 74.2% for the four MSU classification grades. More importantly, although these classification studies provide orthopedists with certain diagnostic evidence, they cannot provide quantitative information about the severity of the disease, and thus have limited roles in relieving the actual workload of orthopedists. To provide a more comprehensive and reasonable evaluation of the degree and region of herniation, we evaluated LDH using 18 categories (combining the severity grades and regions of LDH), four categories (focusing on the severity grades of LDH), and eight categories (focusing on the regions of LDH) for classification. The results indicated that this study not only performed better in terms of accuracy, but also provided more comprehensive quantitative results on the severity of the disease.

To achieve stable results, this study combined segmentation and keypoint detection. In previous studies, Pang et al. [25] successfully applied a segmentation model to achieve precise localization of tissues such as intervertebral discs and articular processes. Meanwhile, Lehnen et al. [26] focused on the application of segmentation models in their research, and accurately detected the range of intervertebral discs on axial lumbar MRI, with an accuracy as high as 87%.

Suri et al. [27] obtained errors of 2.90°, 2.26°, and 3.60° in the measurement of lumbar lordosis angles on MR, CT, and DR images, respectively. In a study by Cina et al. [28] on lumbar DR, they obtained median errors relative to vertebral dimensions, with x and y coordinates of 1.98% and 1.68%, respectively. These studies demonstrate the significant potential of DL models in morphological assessment of medical images. However, to date, there have been no reports on their clinical application.

In light of the aforementioned considerations, in this study we integrated the DL model into the image reading system in a manner that enhanced the efficiency of image reading and the visualization of results, thereby optimizing the process of intervertebral disc region and grade classification. Following rigorous verification on both internal and external test sets, the DL model demonstrated significant effects in improving diagnostic efficiency and accuracy. It can therefore be concluded that the DL model serves as a powerful assistant for clinical orthopedists, assisting them in the recording and observation of disease progression in patients.

Although our study has made positive progress in the automation of LDH diagnosis, there are still certain limitations. Firstly, this retrospective study primarily focused on axial MRIs and did not fully utilize other sources of information such as sagittal MRIs. In the future, we plan to incorporate sagittal MRIs and other relevant imaging data into the analysis to capture richer information features and further optimize the automated diagnostic model. Secondly, the design of a retrospective study itself may not comprehensively reflect the complexity and challenges of the actual clinical diagnostic process. To more robustly validate the clinical utility and efficacy of this diagnostic model, we intend to conduct more rigorous prospective studies in the future to evaluate the model’s performance in a way that is closer to the actual clinical settings.

Finally, we will continue to explore and apply the latest DL algorithms to continuously improve and optimize this diagnostic model. Our goal is to gradually expand its application scope to more diseases of the lumbar spine, such as lumbar spinal stenosis, spondylolisthesis, and vertebral fractures. Through continuous innovation and refinement, we hope to provide orthopedists with a more efficient and accurate aided diagnostic tool, which can help improve treatment outcomes and quality of life for patients.

## Electronic supplementary material

Below is the link to the electronic supplementary material.

Supplementary file 1 (MP4 17632 kb)

## Abbreviations

MRI: Magnetic resonance imaging
LDH: Lumbar disc herniation
CI: Confidence interval
ME: Mean error
P: Precision
F1: F1 score
Kappa: Kappa coefficient
mAP: Mean average precision
SD: Standard deviation
DL: Deep learning
IoU: Intersection over union
SBD: Symmetric bulging disc
CCZ: Central canal zone
LSAZ: Left subarticular zone
LFZ: Left foraminal zone
RFZ: Right foraminal zone
RSAZ: Right subarticular zone
REFZ: Right extraforaminal zone
LEFZ: Left extraforaminal zone
